# A Comparison of Conventional Root Canal Sealers With Ones That Use Green Synthesized Nanoparticles for Antimicrobial Activity: Protocol for a Systematic Review

**DOI:** 10.2196/51351

**Published:** 2024-10-11

**Authors:** Abubaker Mohamed, Enas Ismail, Razia Z Adam

**Affiliations:** 1 Department of Prosthodontics Faculty of Dentistry University of the Western Cape Belville, Cape Town South Africa; 2 Physics Department Faculty of Science (Girl's Branch) Al-Azhar University Cairo Egypt

**Keywords:** root-canal sealers, nanoparticles, antimicrobial activity, root canal, antimicrobial, dentistry

## Abstract

**Background:**

Root canal failure and secondary endodontic infection are frequent clinical scenarios in dentistry. The main microorganisms implicated in root canal therapy failure are persistent *Enterococcus faecalis, Candida albicans,* and *Staphylococcus aureus*. To combat the impact of disease resistance, scientists are concentrating on alternative antimicrobial root canal sealers. Nanomaterials are a recent development in endodontic materials that exhibit great antimicrobial properties, making them an ideal material choice for root canal sealers.

**Objective:**

This systematic review aims to compare the antimicrobial properties of conventional root canal sealers to those incorporating green synthesized nanoparticles between 2010 and 2024.

**Methods:**

A well-constructed protocol was established and registered with PROSPERO (CRD42021286373). Ethics approval was obtained from the Biomedical Research and Ethics Committee from the University of the Western Cape (UWC; BM22/1/4). PRISMA-P (Preferred Reporting Items for Systematic Review and Meta-Analysis Protocols) reporting guidelines were followed. The included criteria demonstrate the green synthesized nanoparticles studies where the nanoparticles (NPs) are incorporated in root canal sealers. MeSH (Medical Subject Headings) terms were used for the search strategy of the systematic electronic databases for articles published in English between 2010 and 2024. The selected databases included Scopus, PubMed, Web of Science, Science Direct, EBSCOhost, SpringerLink, and Wiley Online. A quality assessment tool for laboratory studies will be used to critically appraise the included studies. If applicable, statistical measures (mean, SD, etc) will be used for data analysis and presentation of the results.

**Results:**

The protocol is registered with PROSPERO. A preliminary search was conducted using a determined search strategy across 8 electronic databases, and the review is now complete.

**Conclusions:**

It is anticipated that the results of this systematic review may reveal the increased interest and application for nanoparticle-enhanced root canal sealers. This will aid in the future development of root canal sealants and mitigate the risk of endodontic failure.

**Trial Registration:**

PROSPERO CRD42021286373; https://www.crd.york.ac.uk/prospero/display_record.php?RecordID=286373

**International Registered Report Identifier (IRRID):**

DERR1-10.2196/51351

## Introduction

### Background

In the United States, more than 20 million root canal treatments are completed annually [1]. Worldwide, the international prevalence of people having at least one tooth with a root canal filling is 55.7% (95% CI, 49.6%-61.8%) [2]. Conventional nonsurgical endodontic treatment has a success rate of around 80% [3-5]. However, 22.8% of treated teeth may develop clinical and radiological findings known as apical periodontitis, and 10% of these may require retreatment [6]. The most common reason for endodontic failure is secondary or persistent endodontic infection [7].

Due to numerous and challenging anatomical variances, complete eradication of microbes from the root canal system is not always possible. The root canal system cannot always be completely cleaned of bacteria and their by-products by mechanical and chemical preparation. As a result, an efficient antimicrobial sealer is paramount for a successful root canal treatment [8,9].

In root canal obturation, a variety of sealers with similar properties have been used. Biocompatibility and a potent antimicrobial effect are the main criteria for developing a root canal sealer [10,11]. Conventional root canal sealers exhibit mild to strong antibacterial properties, but once set, the materials possess a diminished antimicrobial capacity [12,13]. Zinc oxide eugenol sealers, for example, have high antibacterial effects at first because of eugenol leaching. However, the antibacterial activity fades with time as eugenol gradually dissipates [14]. A systematic review found that most conventional root canal sealers exhibit strong antibacterial effects immediately after mixing, but these effects gradually decrease and disappear as the sealer hardens over time [13]. The antimicrobial effect of the commercially available bioceramic sealers revealed diverse degrees of antimicrobial capacities of compared sealers [15,16]. A newer root canal sealer with a more potent antimicrobial effect is sought. In recent years, nanomaterials have been used widely in dental materials. Nanoparticles (NPs) are characterized by a small size that is reflected by a large surface area to mass ratio. In addition, its chemical reactivity is improved when compared with the large size of the original material [17,18]. New strategies to improve the success rates of root canal treatment include modifying root canal sealers with strong antimicrobial agents such as nanomaterials [19,20]. Research has shown that the integration of nanomaterials into root canal sealers may result in a drop in the bacterial count penetrating the dentinal tubules [21,22].

### Description of the Secondary Endodontic Infection

The failure of endodontic treatment is defined as the “recurrence of clinical symptoms along with the presence of a periapical radiolucency” [23]. Therefore, clinical presentation and radiographic findings are used to evaluate endodontic failure. The following clinical and radiological criteria are used to diagnose endodontic failure: (1) the presence of clinical signs and symptoms, (2) enlargement of the pre-existing apical radiolucency, (3) formation of a new apical radiolucency related to the tooth, and (4) persistent apical radiolucency related to a tooth that had root canal therapy before 4 years [24]. Failure may occur due to multiple factors, predominantly the presence of persistent intracanal and extracanal microorganisms. Inadequate cleaning and shaping, poor canal obturation, overextension of filling material, poor apical and coronal seals, missed canals without treatment, inadequate access cavity, and some procedural errors such as broken files, ledges, and perforations are other factors contributing to root canal treatment failure [25]. Microbes isolated from teeth undergoing endodontic retreatment include *Enterococcus faecalis, Staphylococcus aureus,* and *Candida albican*s [26-29].

### Description of the Intervention

In biomedical applications, the use of nanotechnology has developed as an encouraging technique for future applications**.** NPs are considered microscopic particles with dimensions ranging from 1 to 100 nm [30]. Different types of NPs have been produced in the last decade. Metal and metal oxide NPs, including silver and zinc oxide, revealed strong antimicrobial effects against endodontic pathogens [31,32]. Furthermore, NPs also exhibit low cytotoxicity to oral tissues [33-35]. In dentistry, NPs are considered effective antimicrobial agents and are superior in comparison to other materials, with a low capacity for bacterial resistance [36].

However, the synthesis of NPs is a complex procedure. There are 2 major pathways for NPs synthesis. The first one is a top-down approach, whereby NPs are synthesized by reducing the size of the desired material from its initial bulk state into smaller particles [37]. Different chemical and physical techniques are used in the reduction, such as mechanical milling, chemical etching, laser ablation, sputtering, thermal ablation, and explosion processes [37]. The second is a bottom-up approach where small structures such as atoms and molecules are joined into larger building blocks and assembled to form the final NPs [37]. Chemical and biological techniques are used in the formation of NPs using the bottom-up approach.

In the biological technique, a green synthesis method involves using plants and their extracts, microorganisms such as bacteria, fungi, actinomyces, and algae [38]. The green synthesis or biosynthesis method is favored over chemical and physical techniques as it is considered to be more environmentally friendly, economical, consumes less amount of energy, biocompatible, and nontoxic [39].

### The Clinical Significance of This Systematic Review

Inflammation of the pulpal tissues and resultant endodontic infection occur when microbes find a convenient portal of entry into the pulpal space [40]. Primary endodontic infection ensues when the microorganisms attack the healthy pulp, causing pathological changes that are followed by pulp necrosis [41]. Secondary endodontic infection is caused when the endodontically treated teeth get reinfected as a result of microorganism invasion into the root canal system [41]. The secondary endodontic infection might happen due to an inadequate coronal seal leading to coronal leakage and intraradicular and extraradicular infection [42,43]. Persistent endodontic infection is observed because of the presence of microorganisms that are part of either primary or secondary endodontic infection. The microorganisms resist the chemo-mechanical preparation as well as the root-filling materials and become a source of persistent infection [44].

This review will have great clinical significance because it will help clinicians choose a more effective root canal sealer for endodontic treatment and identify new areas for dental materials development.

### Aim

This systematic review aims to compare the antimicrobial properties of conventional root canal sealers to those incorporating green synthesized NPs between 2010-2024.

The research question for this systematic review was formulated following the PICO (Population, Intervention, Comparator, and Outcome) format as seen in Table 1: “Do root canal sealers with green synthesized nanoparticles exhibit better antimicrobial efficiency than conventional root canal sealers?”

**Table 1:** PICO^a^ format for the research question.

Frame	Search terms
P (types of sample or participants)	Root canal sealers.
I (types of intervention)	Root canal sealers containing green synthesized NPs^b^.
C (types of control or comparators)	Conventional root canal sealers.
O (type of outcome measures)	Antimicrobial effect or the resolution of infection.

^a^PICO: Population, Intervention, Comparator, and Outcome.

^b^NPs: nanoparticles.

### Objectives

Our objectives are as follows:

- Objective 1: determine the antimicrobial efficacy of 2 root canal sealers (conventional vs those with NPs) in preventing secondary endodontic infection.
- Objective 2: determine the antimicrobial efficacy of root canal sealers with NPs microbiologically.
- Objective 3: determine what other research on root canal sealers with NPs was completed.
- Objective 4: determine how root canal sealers with NPs are prepared (in vitro studies).
- Objective 5: determine how antimicrobial efficacy is investigated.

The goals of this systematic review include gathering research that discusses the use of green synthetic NPs in root canal sealers and comparing their antibacterial efficacy to that of conventional root canal sealers in primary endodontic infection.

## Methods

PRISMA-P (Preferred Reporting Items for Systematic Reviews and Meta-Analysis Protocols; Multimedia Appendix 1) reporting guidelines were followed in this study [45]. A well-constructed protocol was established and registered with PROSPERO (CRD42021286373; Multimedia Appendix 2).

According to this type of study, participants, interventions, outcome measures, and the criteria for selecting studies are described. The setting or context includes any laboratory study where green synthetic NPs were used in root canal sealers.

### Types of Studies

The included studies are laboratory studies (in vitro) conducted between 2010 and 2024, where green synthesized NPs are incorporated in root canal sealers used in endodontics.

### Types of Samples

All in vitro studies, the description of root canal sealers with NPs (material) preparation will be reported.

### Types of Intervention

#### Intervention

Any green synthesized NPs included in root canal sealers.

#### Control

Conventional root canal sealers with no addition of green synthesized NPs.

### Type of Outcome Measures

The outcomes are prespecified and include:

#### Primary Outcome

Resolution of infection with common endodontic microbes with the incorporation of green synthesized NPs in root canal sealers.

#### Secondary Outcome

The antimicrobial activity of root canal sealers modified with NPs can be measured with a variety of methods. These include Kirby Bauer tests, assay tests, direct contact tests, and colony counting. The efficacy of green synthesized NPs on microbes is reported by a clear reduction in colony numbers while measuring the colony-forming units (CFU).

### The Identification of Study Searching Methods: Electronic Searches

A thorough computer search will be carried out for the primary and ongoing studies relevant to the search topic. PubMed, EBSCOhost, Science Direct, Web of Science, Scopus, Wiley Online, BioMed Central, and SpringerLink are the databases that will be used. Studies published between 2010 and 2024 will only be included in their English-language versions. Boolean operators will be used to combine key terms and database-specific functions will be developed to create search strategies for each database. In databases that support this function, MeSH (Medical Subject Headings) terms will be used. Duplicate results from the electronic searches will be eliminated using EndNote (Clarivate) software. Thereafter, title, abstract, and full-text screening will be performed. Searched studies will be managed through Rayyan’s QCRI (Rayyan Systems Inc), an electronic online tool designed to support researchers in conducting systematic reviews.

A comprehensive search strategy focusing on the comparison of the antimicrobial effect of the root canal sealers incorporated with the green synthesized NPs and the conventional root canal sealers will be used. The following is an example of our search strategy: (antibacterial OR antimicrobial) AND (green synthesis OR plant-mediated synthesis OR biosynthesis) AND (nanoparticles OR nanomaterials) AND (root canal sealers OR root canal filling materials) AND (2010-2024).

For further references, all retrieved studies’ reference lists will be observed manually by carrying out meticulous hand-searching for each study. If complete texts are inaccessible, authors will be contacted for the full texts. The PRISMA-P flowchart will be followed in reporting the search results [46].

### Data Collection and Analysis: Study Selection

The data obtained through the database search will be recorded on Rayyan QCRI and subsequently screened for titles and abstracts by 2 review authors (AM and RA) independently. A data extraction sheet will be developed during these phases. The studies found to be relevant or potentially related to the search terms will be obtained and then read and analyzed by 2 different authors (AM and RA) for eligibility criteria. Any disagreement during the 2 phases of screening data will be managed by discussing with a third author (EI).

All selected and screened primary studies that do not meet the eligibility criteria will be excluded and the exclusion reasons will be shared. Secondary studies, including all types of reviews, will be omitted.

### Data Management and Extraction

Using a specifically created prepiloted data extraction form, 2 review authors (AM and RA) will independently collect data on research methodology, participants, interventions, results, and conclusions from each included study. A risk-of-bias evaluation tool will be used by the 2 authors to evaluate the methodological value of all included papers [47]. Results will be reported using Tables and Figures. The retrieved information from all studies will include the following extracted data: (Authors, the title, year of publication, country, study methods, type of materials, type of NPs used, size and concentration of NPs, NPs synthesis routes, the chemicals used, the microorganisms used for antibacterial testing, specimen size, settings, sources of publication, inclusion and exclusion criteria, study methodologies, and statistical analysis). Furthermore, information regarding research funding sources, ethical approval, conclusions, comments, and other necessary correspondence will be retrieved and reported. Any ambiguous or missing data will be reported to the study authors, and any issues will be settled by consensus with the other review authors.

### PRISMA-P

This tool is designed for the standardization of systematic reviews and meta-analysis protocol reporting. Poor systematic review reporting may affect the power and value of the systematic review and will be an unreliable source for clinicians and policymakers. In this systematic review protocol, the PRISMA-P checklist (Multimedia Appendix 1) will be followed. The importance of following PRISMA-P is to ensure all the steps during systematic review construction are strictly respected, this will be done by reporting every single step using the checklist [45].

### Study Quality Assessment: Quality Assessment of Laboratory Studies

For laboratory studies, a critical appraisal tool that was developed by Adam and Khan [47], will be used. In this tool, the following five parameters were used: (1) sampling technique standardization, (2) explanation of sample size estimate, (3) sample calibration in agreement with ISO (International Organization for Standardization) before experimenting, (4) Validity and reliability of outcome measures, and (5) the use of statistical analysis tools (Table 1). Each of the 5 parameters will be scored using a scale from 0-2, as depicted in Table 2. A score of 0 means that the particular parameter is clearly stated in the study. A score of 1 means that the parameter is reported, but it was unclearly reported, or there is a lack of some details regarding the parameter. A score of 2 means the parameter is not reported or hard to find information related to the parameter.

For overall scoring, the total score for each study will be calculated and explained as follows: A score of 0-3 means a low risk of bias. A score of 4-7 means a moderate risk of bias. A score of 8-10 means a high risk of bias for the assessed study; hence, the study is considered of poor quality.

**Table 2:** Quality assessment tool of included laboratory studies [47].

	Assessment criteria	Scores			Total score
		1	2	3	—^a^
1	Sampling technique standardization	—	—	—	—
2	Explanation of sample size estimation	—	—	—	—
3	Sample calibration in agreement with ISO^b^	—	—	—	—
4	Validity and reliability of outcome measures in agreement with ASTM^c^ or ISO	—	—	—	—
5	The uses of proper statistical analysis tools.	—	—	—	—

^a^Not available.

^b^ISO: International Organization for Standardization.

^c^ASTM: American Society for Testing Materials.

### Data Synthesis

If possible, the findings will be compiled, synthesized, and reported in a meta-analysis following a critical evaluation of each of the included studies. When combining data across studies, we will use inverse variance weighting and perform meta-analyses using the DerSimonian and Laird methods. To explore the consistency of effects across studies, we will calculate the *I*² statistic, which quantifies the proportion of total variation attributable to heterogeneity. In addition, we will use Kendall’s τ to assess the relationship between effect sizes and study characteristics. Forest plots will be generated to visually represent the pooled estimates and individual study results. If substantial heterogeneity is detected (*I*²>50%), we will conduct subgroup analyses and meta-regression to investigate potential sources of inconsistency. That will be reported in the included studies. If not, a narrative report will be generated.

#### Managing Missing Data

The authors of the studies will be contacted for any missing data to get any relevant missing data.

#### Assessment of Reporting Biases

In case of publication bias. If possible, a funnel plot will be constructed to report publication bias.

### Ethical Considerations

Ethics approval was obtained from the Biomedical Research and Ethics Committee, University of the Western Cape (UWC; BM22/1/4).

## Results

**Figure 1:**
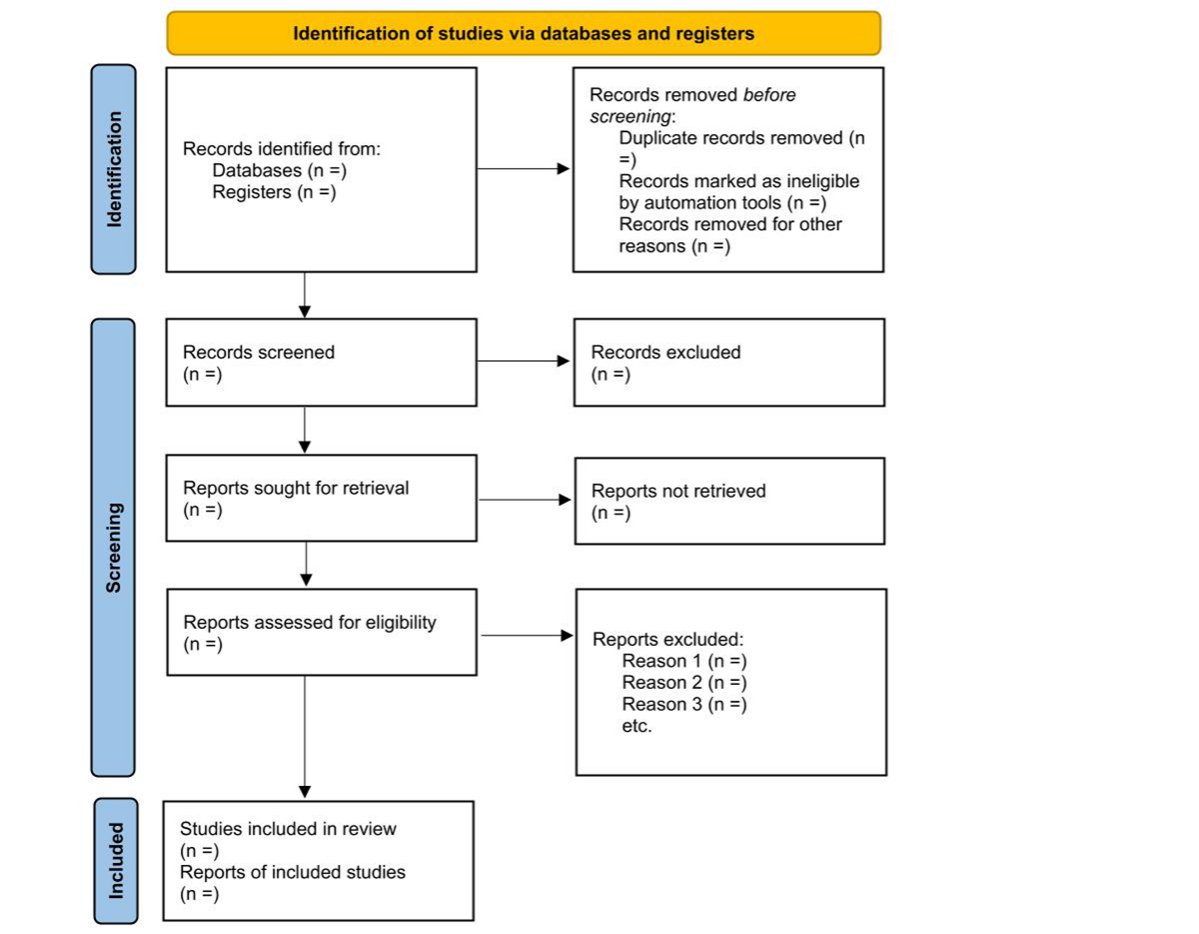
PRISMA (Preferred Reporting Items for Systematic Reviews and Meta-Analyses) 2020 flowchart.

This systematic review protocol, as seen in Figure 1, aims to compare the biological activity of root canal sealers incorporated with nanoparticles to the conventional ones. Following the use of preset inclusion criteria, a preliminary search will be conducted using a determined search strategy across the databases, the final laboratory studies will be identified, and the data will be extracted. If possible, a meta-analysis will be constructed, or a narrative presentation of the findings will be finalized. The review process is expected to be completed by December 2024. The systematic review findings will be disseminated and submitted for publication in a peer-reviewed journal.

## Discussion

This systematic review will comprehensively look at the antimicrobial activity of root canal sealers incorporated with green synthesized nanoparticles compared with conventional root canal sealers. We anticipated that the use of green synthesized NPs could enhance the antimicrobial activities of root canal sealers. In the literature, the incorporation of chemically or physically synthesized nanoparticles in root canal sealers revealed superior biological properties [11,48,49]. Thus, the findings of this systematic review may highlight the significance of green synthetic nanoparticles in endodontics.

Evidence-based practice is formed by the assimilation of good-quality clinical knowledge and patient values mixed with the most excellent research evidence. Systematic reviews are secondary research used to detect, appraise, and recapitulate the findings of the primary research, including laboratory and clinical trials, in order to inform the decision makers about health care–related topics. Thus, systematic review findings can be interpreted and implemented in clinical settings [50]. Conducting systematic reviews is important as it synthesizes data from many studies into 1 report [51]. Up-to-date research knowledge regarding prognostic factors, diagnostic tests, and interventions is gained by conducting precise, systematic reviews [52].

Laboratory studies involving dental materials are not often transformed into clinical trials. Commonly, the high cost of these in vitro studies means a significant financial investment is required. In addition, the production of feasible clinical research outcomes is doubtful as the translation of laboratory findings into clinical research may take longer than expected due to ethical issues. Long follow-up and observation periods during clinical trials may also play a role. The translation of laboratory findings into clinical studies is primarily caused by all the aforementioned problems.

The state of the included studies and the strength of their findings will determine whether this systematic review will have an impact on the investigator’s choice regarding the use of nanomaterials in endodontics.

The anticipated limitation of this systematic review is mainly around the type of included studies. Since the subject of the search is entirely novel to the field of endodontics, there are not likely to be enough clinical studies using the necessary intervention. High-quality clinical trials with rigorous designs and a controlled setting are required for the conversion of knowledge from research into clinical practice. Furthermore, it is argued that the comparability of laboratory investigations is significantly impacted by the lack of procedure standardization across various study designs.

Another restriction is the usage of many databases for searching; each database has its specific constraints and functionality. It is not always possible to use the same search string across all databases; some databases may not accept long search keywords. Furthermore, the different search engines use various sets of filters. In conclusion, the use of green synthesized NPs in endodontic sealers is still new. Thus, the expected transformation of laboratory studies into clinical trials will not be enough to be included in this systematic review.

### Conclusion

In conclusion, this systematic review protocol aims to provide a comprehensive evaluation of the antimicrobial properties of endodontic sealers incorporated with green synthesized NPs compared with conventional root canal sealers. Using robust techniques, such as specific inclusion and exclusion criteria and using the suitable search string across various databases, leads to the inclusion of relevant laboratory and clinical studies. Finding out the quality of the acquired data will be made easier by using the proper critical appraisal tool. This review will provide useful insights into the possible advantages of using green synthesized NPs in endodontic treatment and enhance clinical practice by gathering available information and critically evaluating study methods.

## Appendix

Multimedia Appendix 1 PRISMA-P (Preferred Reporting Items for Systematic Review and Meta-Analysis Protocols) checklist.

Multimedia Appendix 2 Prospero.

## Abbreviations

CFU: colony-forming unit
ISO: International Organization for Standardization
MeSH: Medical Subject Headings
NP: nanoparticle
PICO: Population, Intervention, Comparator, Outcome
PRISMA-P: Preferred Reporting Items for Systematic Review and Meta-Analysis Protocols
UWC: University of the Western Cape

## Data Availability

The data sets generated during and/or analyzed during this study are available from the corresponding author on reasonable request.
