# Supplementary material for: A Comparison of Conventional Root Canal Sealers With Ones That Use Green Synthesized Nanoparticles for Antimicrobial Activity: Protocol for a Systematic Review
Source: JMIR Res Protoc. 2024 Oct 11;13:e51351. doi: 10.2196/51351 (PMC11512129; doi:10.2196/51351)
Supplement: Multimedia Appendix 2 [file resprot_v13i1e51351_app2.pdf]

A comparison of effectiveness of nanoparticle modified endodontic sealers and conventional endodontic sealers.

To enable PROSPERO to focus on COVID-19 submissions, this registration record has undergone basic automated checks for eligibility and is published exactly as submitted. PROSPERO has never provided peer review, and usual checking by the PROSPERO team does not endorse content. Therefore, automatically published records should be treated as any other PROSPERO registration. Further detail is provided here.

Citation

Razia Adam, Abubakr Mohamed, Suwayda Ahmed. A comparison of effectiveness of nanoparticle modified endodontic sealers and conventional endodontic sealers.. PROSPERO 2021 CRD42021286373 Available from:
https://www.crd.york.ac.uk/prospero/display_record.php?ID=CRD42021286373

Review question

In patients with irreversible pulpitis requiring root canal treatment, do root canal sealers with nanoparticles exhibit better antimicrobial efficiency than conventional root canal sealers?

Searches

The following search strategy will be used in PubMed/MEDLINE and modified for the other databases:

• (patient* with irreversible pulpitis OR adult* with irreversible stomatitis) AND root canal treatment AND (endodontic seal*ers OR root canal seal*ers) AND nanoparticles AND (antibacterial OR antimicrobial).

• Database:

Electronic database includes, PubMed, Scopus, MEDLINE, Central, Embase, Web of Science, ScienceDirect. Google Scholar, ISI Citation Indices, and Web of Science ISI proceedings (conference proceedings). This will be complemented by hand-searching the reference lists of the selected studies for additional relevant studies

Types of study to be included

In vitro studies, in vivo, human, and animal studies,

Condition or domain being studied

Pulpal and peri-apical tissues remain healthy until they have been exposed to prolonged irritation. Pulpal and peri-apical inflammation is considered a final outcome of long-standing exposure to pulpal irritant. Bacteria and bacterial products are the main causative factors for pulpal inflammation and failure of root canal therapy. The primary pulpal infection followed by pulpal necrosis is initiated primarily by 2 bacteria Porphyromonas Gingivalis and Porphyromonas Endodontalis . In failed root canal treatments and secondary pulpal infection, the main micro-organisms are Enterococcus faecalis and Candida albicans, due to their strong features and resistant nature. Complete removal of micro-organisms from the root canal system is not always achieved due to different and difficult anatomical variations. Mechanical preparation using files followed by chemical preparation are not always successful in eliminating all the microorganisms and by-products in the root canal system. Therefore, there is a need for an effective antimicrobial sealer to augment eradication of residual bacteria . Antimicrobial sealers also aid the apical and peri-apical tissues to repair and restore it’s normal function as well as inhibiting microbial proliferation .

Participants/population

Population • Patients with Asymptomatic irreversible pulpitis; requiring root canal treatment • Patients with reversible pulpitis; not requiring root canal treatment

Intervention • Root canal sealers with nanoparticles • Root canal sealers without nanoparticles

Comparison • Conventional root canal sealers • Root canal sealers containing nanoparticles or non-root canal sealers material

Outcome • Antimicrobial effect against Endodontic pathogens (Enterococcus faecalis, Candida albicans, etc…) • Other outcomes rather than antimicrobial effect

Intervention(s), exposure(s)

C. Types of interventions:

I. Intervention

Any nanoparticles synthesised and included into endodontic sealers

II. Control

Endodontic sealers with no modifications

D. Types of outcome measures

The outcomes pre-specified and that will be used for this Overview include:

I. Primary outcomes

The efficacy / antimicrobial activity of nanoparticles in endodontic sealers.

Comparator(s)/control

Conventional endodontic sealers and nanoparticle modified endodontic sealers modified.

Main outcome(s)

The efficacy / antimicrobial activity of nanoparticles in endodontic sealers.

Additional outcome(s)

not applicable

Data extraction (selection and coding)

The data obtained via database search will be screened for titles and abstracts by two different authors independently. The studies found to be relevant or potentially related to the search terms will be obtained and then read and analysed by two different authors for eligibility criteria. Any disagreement during the two phases of screening data will be managed by discussion between the two authors. After both phases of screening data extraction sheet will be made and then tested on two studies and then refined according to the trial. Two different authors will extract data, any disagreement on data extraction will be solved by further discussion between the two authors.

• Data extraction and management:

Two review authors (AA and RA) will independently extract information on study methods, participants, interventions, outcomes, and conclusions from each included SR using a specially designed pre-piloted data extraction form.

The risk of bias of each included study to assess the methodological quality of the included study will independently be completed by 2 reviewers. The extracted data will be recorded on the Table of Characteristics of included studies. Information retrieved from the included studies will also include authors, titles, country, setting, source of publication, number of patients studied, patient demographic characteristics, response rate, inclusion and exclusion criteria of participants, study methods utilised, and statistical analyses. In addition, funding sources of projects, ethical clearance, conclusions, comments, and correspondence required will also be extracted. Study authors will be contacted in the case of unclear or missing data and any disagreements will be resolved by consensus with the other review authors.

Risk of bias (quality) assessment

All selected studies will be analysed and assessed for the risk of bias using the Cochrane risk of bias tool(37).

Strategy for data synthesis

The results, after critical appraisal of all the included studies will be collated, synthesised, and reported in a meta-analysis, if possible. If not, it will be reported in the form of a narrative.

Analysis of subgroups or subsets

not applicable

Contact details for further information

Razia Adam

rzadam@uwc.ac.za

Organisational affiliation of the review

University of the Western Cape

Review team members and their organisational affiliations

Dr Razia Adam. University of the Western Cape

Dr Abubakr Mohamed. UWC

Dr Suwayda Ahmed. UWC

Collaborators

Assistant/Associate Professor Fanelwa Ajayi. UWC

Type and method of review

Meta-analysis, Narrative synthesis, Systematic review

Anticipated or actual start date

24 January 2022

Anticipated completion date

15 December 2022

Funding sources/sponsors

None applicable.

Conflicts of interest

Language

English

Country

South Africa

Stage of review

Review Ongoing

Subject index terms status

Subject indexing assigned by CRD

Subject index terms

Enterococcus faecalis; Humans; Nanoparticles; Root Canal Filling Materials

Date of registration in PROSPERO

20 November 2021

Date of first submission

20 October 2021

Details of any existing review of the same topic by the same authors

Adam RZ, Khan SB (2021) Antimicrobial efficacy of silver nanoparticles against Candida albicans: A systematic review protocol. PLoS ONE 16(1): e0245811. https://doi.org/10.1371/journal.pone.0245811

Stage of review at time of this submission

The review has not started

Stage	Started	Completed
Preliminary searches	No	No
Piloting of the study selection process	No	No
Formal screening of search results against eligibility criteria	No	No
Data extraction	No	No
Risk of bias (quality) assessment	No	No
Data analysis	No	No

The record owner confirms that the information they have supplied for this submission is accurate and complete and they understand that deliberate provision of inaccurate information or omission of data may be construed as scientific misconduct.

The record owner confirms that they will update the status of the review when it is completed and will add publication details in due course.

Versions

20 November 2021

20 November 2021
